# Pilot assessment of probiotics for pregnant women in Rwanda

**DOI:** 10.1371/journal.pone.0195081

**Published:** 2018-06-18

**Authors:** Amy McMillan, Stephen Rulisa, Gregory B. Gloor, Jean M. Macklaim, Mark Sumarah, Gregor Reid

**Affiliations:** 1 Centre for Human Microbiome and Probiotics, Lawson Health Research Institute, London, Canada; 2 Department of Cellular and Molecular Medicine, Lerner Research Institute, Cleveland Clinic, Cleveland, United States of America; 3 Department of Obstetrics and Gynecology, University of Rwanda, Kigali, Rwanda; 4 Department of Biochemistry, and Applied Mathematics, The University of Western Ontario, London, Canada; 5 Agriculture and Agri-Food Canada, London, Canada; 6 Department of Microbiology and Immunology, The University of Western Ontario, London, Canada; University of Cape Town, SOUTH AFRICA

## Abstract

**Background:**

While the global market for probiotics is soon to reach in excess of US$50 billion, the continent of Africa has been largely ignored, despite these products having the ability to reduce the burden of disease and death.

**Trial design:**

The present randomised, blinded, placebo-controlled clinical trial was undertaken in Rwanda, a country devoid of well-documented probiotics. The primary outcome aim was to examine receptivity and compliance for orally administered probiotic capsules containing *Lactobacillus rhamnosus* GR-1 and *Lactobacillus reuteri* RC-14 in pregnant women and assess any initial side effects or changes to the vaginal microbiome.

**Methods:**

Pregnant women between the ages of 18 and 55 were recruited from the Nyamata District Hospital in Rwanda and randomly assigned to receive probiotic or placebo capsules for one month. Clinicians were blinded to the treatments.

**Results:**

The drop-out rate was 21%, with 13 of 18 women in the placebo group and 17 of 20 in the probiotic group completing the study. Only 13 women returned for birthing and additional sample collection. No side effects of either treatment group were reported. Microbiota and metabolomics data showed similar findings to those reported in the literature, with low bacterial diversity and *Lactobacillus* dominance associated with a healthy vagina, and birthing associated with high diversity. Despite the small sample size and lack of changes in the microbiota, women in the placebo arm were significantly more likely to give birth pre-term.

**Conclusion:**

Overall women were receptive to the probiotic concept, but the lack of information on such products and logistical and economical challenges pose problems for wider population engagement.

**Trial registration:**

ClinicalTrials.gov NCT02150655

## Introduction

Over the past twenty or so years, probiotic (live organisms that when administered in adequate amounts confer a healthy benefit in the host) sales have skyrocketed around the developed world. This has been partly fostered by the growing scientific and clinical evidence of their usefulness in treating and preventing diseases [1–4]. The efficacy against urogenital, diarrheal and respiratory diseases are particularly important for women and children in developing countries, where preventable morbidity and mortality occurs [5–7].

In Rwanda, a developing country with female life expectancy of 59.7 years, 4.1 live births per woman [8], and extremely high rates of exclusive breast feeding [9], health care is delivered through district hospitals and a growing number of clinical centres. The high retention of HIV-infected women and infants to continue with their care suggests a willingness to comply if healthcare products are available, affordable and accessible [10].

Through a long-standing collaboration with Rwandan physicians, we conducted a study aimed at identifying biomarkers of bacterial vaginosis (BV), a condition characterized by increased bacterial diversity of the vaginal microbiota [11]. BV is the most prevalent vaginal condition and is associated with increased risk of HIV transmission and preterm labor, in addition to unpleasant symptoms such as malodo2 [12]. Previous studies by our group have demonstrated that the probiotic *L*. *rhamnosus* GR-1 combined with *L*. *reuteri* RC-14 is effective in restoring a *Lactobacillus-*dominated microbiota in women with BV when combined with antimicrobial therapy [13]. We hypothesized that this probiotic could help maintain a healthy vaginal microbiota in Rwandan pregnant women. The aim of the present study was to introduce the concept of probiotics to pregnant women in Rwanda, assess safety and receptivity with one month of treatment at various gestational times, and examine whether the therapy impacted the vaginal microbiota.

## Materials and methods

### Study design

Healthy pregnant women between the ages of 18 and 55 and a gestational age less than 36 weeks were recruited from the Nyamata District Hospital in Rwanda between September and December, 2012. The Health Sciences Research Ethics Board at Western University, Canada, and the CHUK Ethics Committee, Rwanda granted ethical approval for all experiments involved in the study. The methods were carried out in accordance with the approved guidelines and all women provided written informed consent.

Subjects were stratified by malaria exposure (past malaria, present malaria or malaria naïve) and treatment (probiotic or placebo) was randomly assigned within each group by hospital pharmacy. None of the authors had a role in subject assignment. None of the subjects or relatives were aware of their regimen until post-study. Participants were excluded if they had a current infection of gonorrhoea, *Chlamydia*, genital warts, active genital herpes lesions, active syphilis, urinary tract infections, were receiving drug therapy that may affect the vaginal microbiome, had unprotected sexual intercourse within the past 48 hours, used a vaginal douche, genital deodorant or genital wipe in past 48 hours, or had taken any probiotic supplement in past 48 hours. After reviewing details of the study, participants gave their signed consent before enrolment. They were randomly assigned by numbered containers to received either one gelatin capsule containing one billion each of *Lactobacillus rhamnosus* GR-1 and *L*. *reuteri* RC-14 or visually identical calcium carbonate placebo (Chr Hansen, Denmark) by mouth each day for one month.

### Sampling

For metabolome analysis, sterile Dacron polyester-tipped swabs (BD) were pre-cut with sterilized scissors and weighed in 1.5 ml microcentrifuge tubes prior to sample collection. Using sterile forceps to clasp the pre-cut swabs, a nurse obtained vaginal samples for metabolomic analysis by rolling the swab against the mid-vaginal wall. A second full-length swab was obtained for Nugent Scoring and 16S rRNA gene sequencing using the same method. Vaginal pH was measured using pH strips. Samples were frozen within 2 hours of collection and stored at -20°C or below until shipment to Canada for analysis. Malaria status was assessed using the CareStart^TM^ Malaria Rapydtest® kit (Diasys, Berkshire, UK). Sampling was conducted at recruitment (Visit 1), after 1 month of treatment (Visit 2), and again at birth (birth). Samples were coded and the patient identification only released upon completion of microbiome and metabolomic analyses.

### Microbiome profiling

Vaginal swabs for microbiome analysis were extracted using the QIAamp DNA stool mini kit (Qiagen) with the following modifications: swabs were vortexed in 1 mL buffer ASL before removal of the swab and addition of 200 mg of 0.1 mm zirconia/silica beads (Biospec Products). Samples were mixed vigorously for 2 x 30 seconds at full speed with cooling at room temperature between (Mini-BeadBeater; Biospec Products). After heating to 95°C for 5 minutes, 1.2 ml of supernatant was aliquoted into a 2ml tube and one-half an inhibitEx tablet (Qiagen) was added to each sample. All other steps were performed as per the manufacturers’ instructions. Sample amplification for sequencing was carried out using the forward primer (ACACTCTTTCCCTACACGACGCTCTTCCGATCTnnnn(8)CWACGCGARGAACCTTACC) and the reverse primer (CGGTCTCGGCATTCCTGCTGAACCGCTCTTCCGATCTn(12)ACRACACGAGCTGAC GAC) where nnnn indicates four randomly incorporated nucleotides, and (8) was a sample nucleotide specific barcode. The 5’ end is the adapter sequence for the Illumina MiSeq sequencer and the sequences following the barcode are complementary to the V6 rRNA gene region. Amplification was carried out in 42 μL with each primer present at 0.8 pMol/mL, 20 μL GoTaq hot start colorless master mix (Promega) and 2 μL extracted DNA. The PCR protocol was as follows: initial activation step at 95°C for 2 minutes and 25 cycles of 1 minute 95°C, 1 minute 55°C and 1 minute 72°C.

All subsequent work was carried out at the London Regional Genomics Centre (LRGC, lrgc.ca, London, Ontario, Canada). Briefly, PCR products were quantified with a Qubit 2.0 Flourometer and the high sensitivity dsDNA specific fluorescent probes (Life Technologies). Samples were mixed at equimolar concentrations and purified with the QIAquick PCR Purification kit (QIAGEN). Samples were paired-end sequenced on an Illumina Mi-Seq with the 600-cycle version 3 reagents with 2x220 cycles. Data were extracted from only the first read, since it spanned the entirety of the V6 region including the reverse primer and barcode.

Resulting reads were extracted and de-multiplexed using modifications of in-house Perl and UNIX-shell scripts with operational taxonomic units (OTUs) clustered at 97% identity, similar to our reported protocol. Automated taxonomic assignments were carried out by examining best hits from comparison the Ribosomal Database Project (rdp.cme.msu.edu) and manually curated by comparison to the Green genes database (greengenes.lbl.gov) and an in-house database of vaginal sequences (Macklaim unpublished). Taxa with matches at least 95% identity to query sequences were annotated as such. OTUs were summed to the genus level except for lactobacilli, and rare OTUs found at less than 0.5% abundance in any sample removed in all samples. To control for background contaminating sequences, a no-template control was also sequenced. Barplots were constructed with R Core Team using proportional values.

### Statistical analyses

To avoid inappropriate statistical inferences made from compositional data, centred log-ratios (clr), a method previously described by Aitchison [14] and adapted to microbiome data were used with paired t-tests for comparisons of genus and species level data [15,16]. The Benjamini Hochberg False Discovery rate (FDR) method was used to control for multiple testing with a corrected p value threshold of 0.1 [17]. All statistical analysis, unless otherwise indicated, was carried out using R version 3.2.4 (r-project.org, R foundation for statistical computing). A one-way repeated measures ANOVA was used to assess the relationship between bacterial diversity (Shannon’s Diversity Index (H)) and visit followed by pairwise t-tests with FDR corrections to identify significant pairs.

### Sample preparation GC-MS

Vaginal swabs were pre-cut into 1.5 mL tubes and weighed prior to and after sample collection to determine the mass of vaginal fluid collected. After thawing, swabs were eluted in methanol-water (1:1) in 1.5 mL microcentrifuge tubes to a final concentration of 50 mg vaginal fluid/mL, which corresponded to a volume ranging from 200–2696 μL, depending on the mass of vaginal fluid collected. A blank swab eluted in 800 μL methanol-water was included as a negative control. All samples were vortexed for 10 s to extract metabolites, centrifuged for 5 min at 10 621 g, vortexed again for 10 s after which time the brushes were removed from tubes. Samples were centrifuged a final time for 10 min at 10 621 g to pellet cells and 200 μL of the supernatant was transferred to a GC-MS vial. The remaining supernatant was stored at -80°C for LC-MS analysis. Next, 2 μL of 1 mg/mL ribitol was added to each vial as an internal standard.

Samples were then dried to completeness using a SpeedVac. After drying, 100 μL of 2% methoxyamine-HCl in pyridine (MOX) was added to each vial for derivatization and incubated at 50°C for 90 min. 100 μL N- Methyl-N-(trimethylsilyl) trifluoroacetamide (MSTFA) was then added and incubated at 50°C for 30 min. Samples were then transferred to micro inserts before analysis by GC-MS (Agilent 7890A GC, 5975 inert MSD with triple axis detector). One μL of sample was injected using pulsed splitless mode into a 30 m DB5-MS column with 10 m duraguard, diameter 0.35mm, thickness 0.25 μm (JNW Scientific). Helium was used as the carrier gas at a constant flow rate of 1 ml/min. Oven temperature was held at 70°C for 5 min then increased at a rate of 5°C/min to 300°C and held for 10 min. Solvent delay was set to 13 min to avoid solvent and a large lactate peak, and total run time was 61 min. Masses between 25 m/z and 600 m/z were selected by the detector. All samples were run in random order and a standard mix containing metabolites expected in samples was run multiple times throughout to ensure machine consistency.

### Data processing GC-MS

Chromatogram files were deconvoluted and converted to ELU format using the AMDIS Mass Spectrometry software, with the resolution set to high and sensitivity to medium. Chromatograms were then aligned and integrated using Spectconnect [18], with the support threshold set to low. All metabolites found in the blank swab, or believed to have originated from derivatization reagents were removed from analysis at this time. After removal of swab metabolites, the IS matrix from Spectconnect was transformed using the additive log ratio transformation (alr) [14] and ribitol as a normalizing agent (log2(x) / log2(ribitol)). Zeros were replaced with two thirds the minimum detected value on a per metabolite basis prior to transformation. All further metabolite analysis was performed using these alr transformed values.

Metabolites were initially identified by comparison to the NIST 11 standard reference database (http://www.nist.gov/srd/nist1a.cfm). Identities of metabolites of interest were then confirmed by authentic standards if available. Principal component analysis (PCA) was performed in R using the FactoMineR package with pareto scaling [19].

## Results

**Fig 1:**
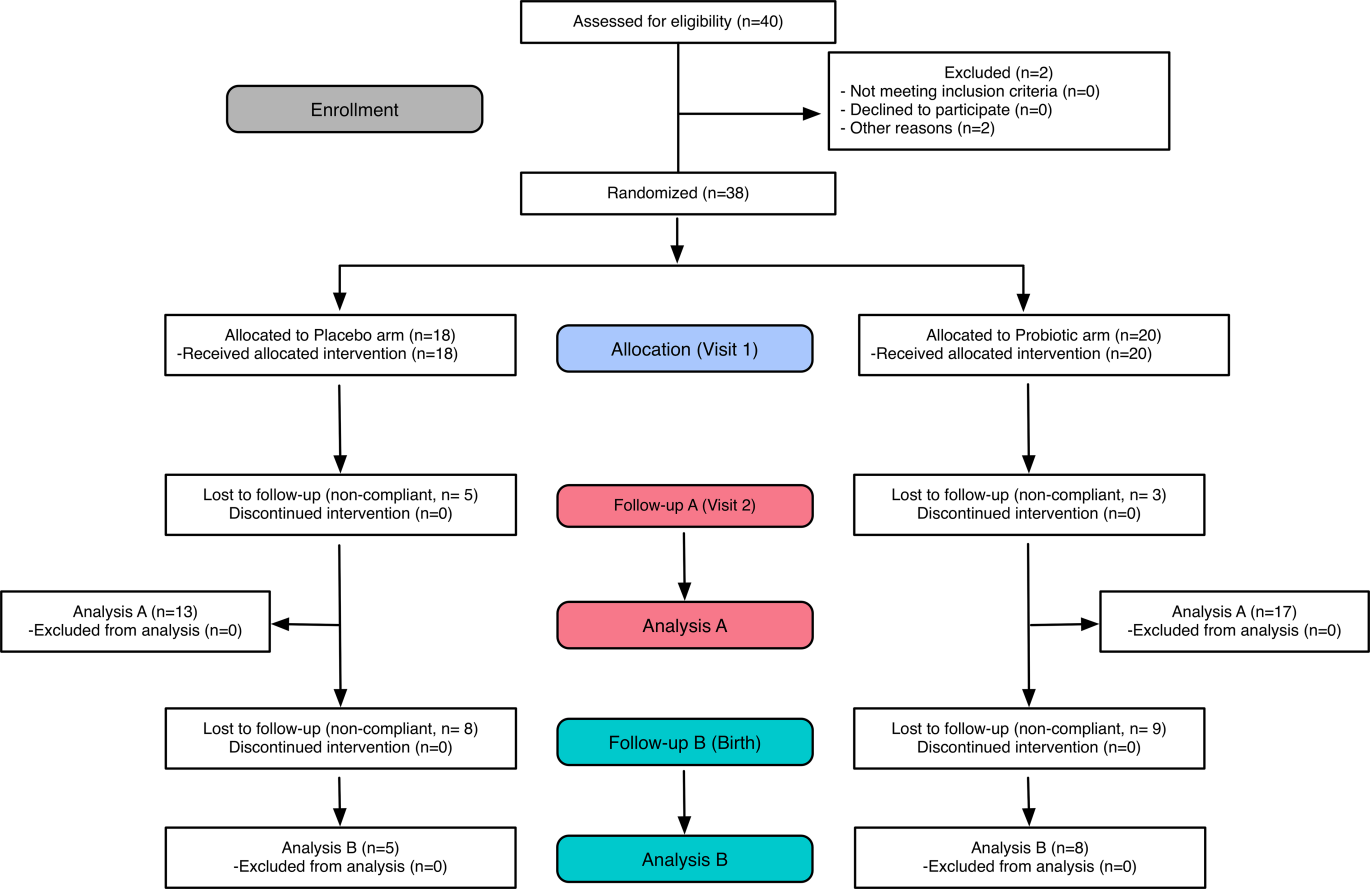
Flowchart of study design.

The mean age of the 20 women who received probiotics and 18 women who received placebo for one month was 26.3 and 27.6 years respectively (Wicoxon test, P>0.05). Gestational age differed significantly between the two groups (Wicoxon test, P = 0.03) with that of the probiotic group being 17 weeks (range 4 to 28), and 22 weeks (range 8–32) for women receiving placebo. Within the placebo group, 4 subjects (22%) currently had malaria, 7 (39%) had malaria in the past, and 7 (39%) had never had malaria. In the probiotic group, 5 subjects (25%) currently had malaria, 8 (40%) had malaria in the past, 6 (30%) had not had malaria previously, and 1 had no data available. Of the 20 subjects on probiotics, 17 (85%) were compliant for the full one month of treatment; of 18 on placebo, 13 (72%) were fully compliant (Fig 1). While compliance relies on patient honesty, the subjects who returned were motivated and gave what staff perceived to be honest responses. No single reason was identified for this lack of compliance, but inconvenience to the clinic, lack of any clinical problems, not wishing to report on capsule use, lack of contact information (some subjects did not provide phone numbers) were perceived by the staff. Of those who completed the study, no issues were raised about side effects or concerns over the capsule intake. Patients showed an interest in learning more about the probiotics and considering them as part of a health strategy, with neither excess enthusiasm or extreme concern was evident.

Of the 13 women who were present for birthing (43.3% of women who completed visit 2), 5 were in the placebo group and 8 in the probiotic group. The reason for not all women delivering at the hospital was preferred delivery at home or at a local clinic. Women in the placebo group were significantly more likely to give birth preterm (Fisher’s exact test P = 0.045), with three women in the placebo group experiencing preterm labor (25 weeks (deceased), 33 weeks, and 36 weeks) compared to none in the probiotic group.

**Fig 2:**
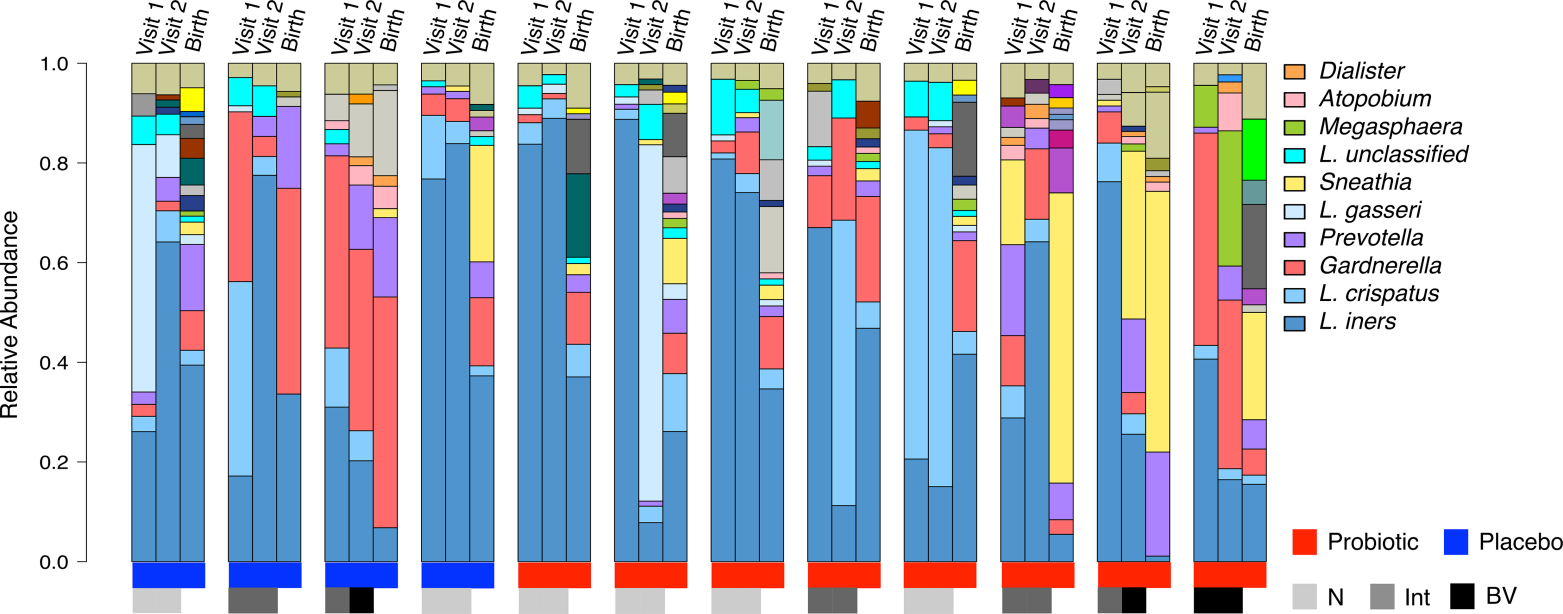
Vaginal microbiota at recruitment, after 1 month probiotic or placebo and at birth. Only subjects who completed the study to birth visits are shown (N = 12). One subject in the placebo group was unable to provide a sample at birth and therefore is not included in the above figure. Nugent scores were not collected for birth visits. N: Normal Nugent Score, Int: Intermediate Nugent Score, BV: Bacterial Vaginosis Nugent Score.

Bacterial vaginosis was not prevalent in either group at visits 1, 2, or 3. In the placebo group, there were no cases detected by Nugent Gram stain scoring [20] at visit 1, and three detected at visit 2; while three women in the probiotic group were positive for BV at visit 1, and six at visit 2. Of the women who completed the study and gave birth at our clinic, there was no significant difference in any taxa after one-month probiotic or placebo treatment (FDR corrected P > 0.1), nor were there any significant changes in Nugent scores due to probiotic treatment (Fig 2). *Lactobacillus iners*, *L*. *crispatus* and *L*. *gasseri* were the most abundant *Lactobacillus* species. Only one woman in the probiotic group had malaria at 1 month follow-up visit (visit 2).

**Fig 3:**
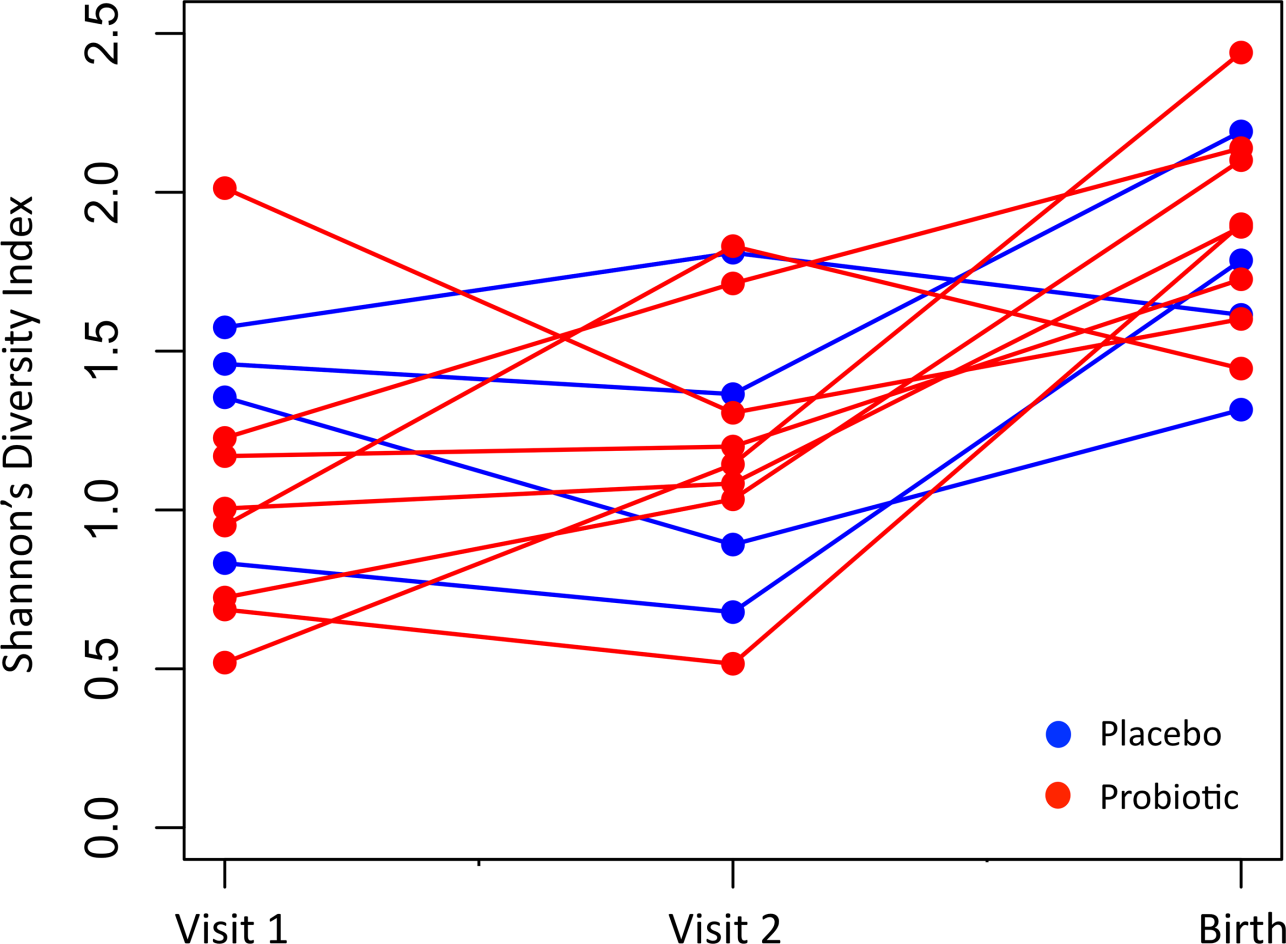
Shannon’s diversity Index of the vaginal microbiota of women at recruitment (Visit 1), after one month (Visit 2) and within one month after birth. Each line represents a single woman over time. Only subjects who completed the study to birth visits are shown (N = 12).

The diversity of the vaginal microbiota was significantly higher at birth than at visit 2 (paired t-tests, FDR corrected P = 0.004, Fig 3), with ten of the women showing an increase in mean diversity at birth compared to the previous visit, and only two showing a decrease.

**Fig 4:**
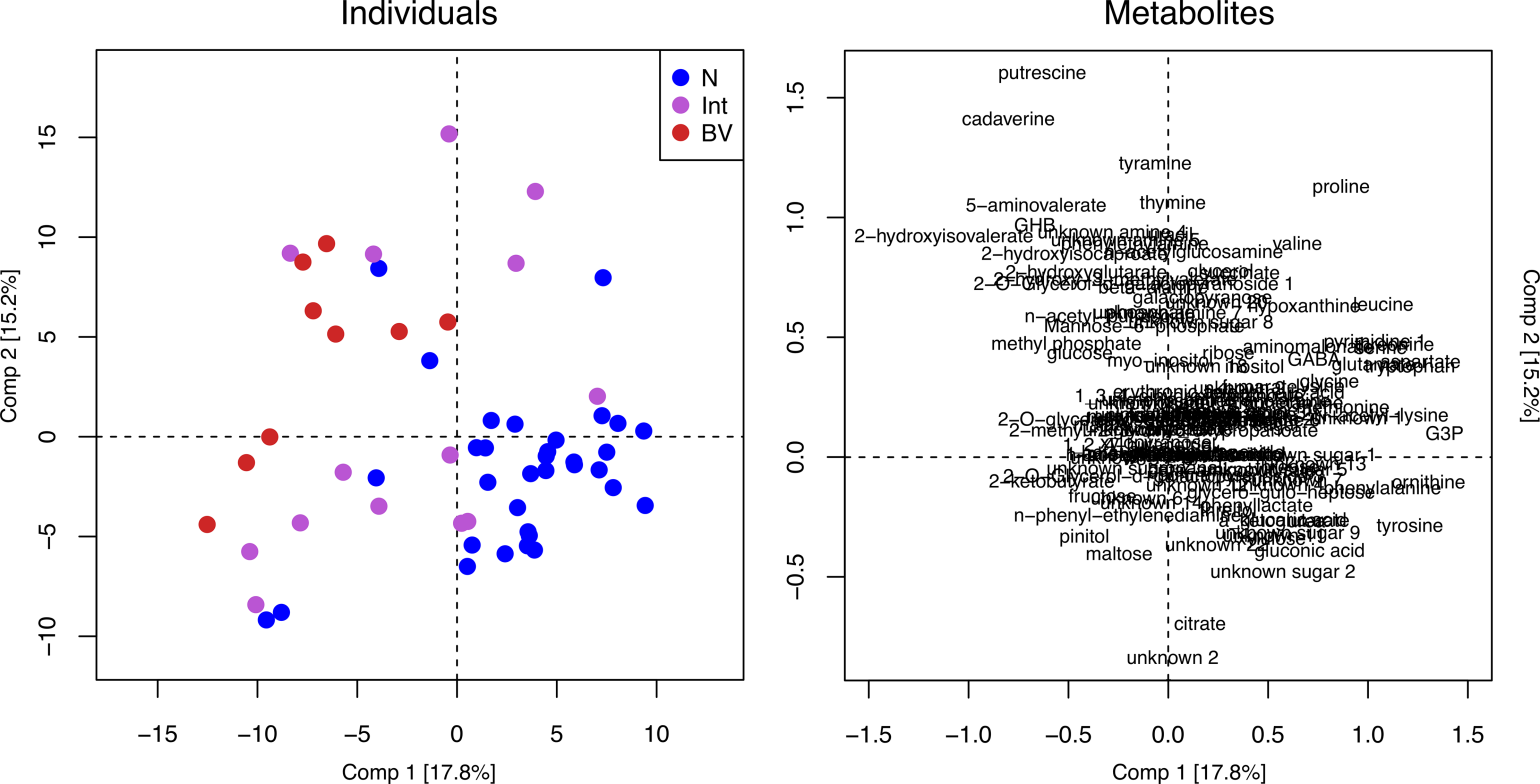
Principal component analysis (PCA) plots built from 128 metabolites detected by GC-MS. Each point on the scoreplot on the left represents a single sample. The loadings plot on the right depicts metabolites. Points are colored according to the Nugent scoring criteria for diagnosis of bacterial vaginosis (BV), where N = Normal, Int = intermediate and BV = bacterial vaginosis.

The vaginal metabolome of women with BV was distinct from healthy women with intermediate Nugent score samples clustering equally with BV or Normal samples (Fig 4). The most obvious metabolite differences were the same as observed in our previous study [11].

## Discussion

This is the first study of encapsulated probiotics for pregnant women in Rwanda. Despite being administered orally at various times of gestation, including as early as week 4, no side effects were reported by the subjects. We cannot discount side effects being the reason for subjects who did not return for visit two, but it was not logistically possible to follow up these women in their rural communities.

We did not observe any significant differences in taxa between women on the probiotic compared to placebo treatment. This lack of findings may be due to insufficient power resulting from small sample sizes. A similar study by our group in Brazilian women found significant increases in cure rates for BV as well as decreased bacterial diversity following probiotic treatment for a one-month period [13,21]. Disparities in results may be due to the fact that women in the Brazilian study were not exclusively pregnant and the dose was double what was employed in the current study. The sample size was also much larger, with 31 women with BV enrolled in each arm (probiotic vs placebo). It is also possible that a much longer duration of probiotic therapy may be needed to potentially impact the vaginal microbiota, assuming the strains do have an impact. Despite the small number of women who completed the study to birth (N = 13), there was a statistically significant difference in preterm labor rates between placebo and controls, with 60% of the placebo group giving birth preterm compared to 0% of the probiotic group. A larger study is justified to validate these findings.

Compliance was high amongst this group of women for the duration of treatment, with only 15% drop out in the probiotic arm. The reason for the higher drop-out rate for the placebo group (28%) was not determined. As it was not logistically possible to follow up women in rural communities, we cannot determine if side effects contributed at all to drop outs.

The vaginal bacterial profiles were remarkably similar to those reported in studies of women from other continents [22–25].^.^ This further suggests a universality in which only four or five species of *Lactobacillus* dominantly colonize the host irrespective of race, diet and lifestyle [24]. As with other countries, high bacterial diversity was associated with BV, with the same groups of anaerobic bacteria that produce a range of metabolic markers for dysbiosis and the presence of malodor [11,26,27]. We also observed a significant increase in bacterial diversity after birth, again consistent with recent findings [28]. The evolutionary purpose for this shift in community composition remains unknown.

While the study provided no clinical reason for probiotics not to be used by women in Rwanda, the issue of product cost does need to be discussed. Thirty capsules of *Lactobacillus* GR-1 and RC-14 retail for around $36 in Canada. This puts it far beyond the majority of Rwandans whose average salary is US$13,614 per annum [29]. It emphasizes the need for a different business model in order to create affordable probiotics for developing countries like Rwanda. It is with this goal in mind, that the *Lactobacillus* GR-1 strain (and a generic version of *Lactobacillus rhamnosus* GG) has been developed in sachet form with *Streptococcus thermophilus* with a two-year shelf-life at room temperature for use in creating probiotic yogurt in east Africa [30]. This is made possible by a donation of the strain by Chr Hansen who own the rights to the GR-1 strain. The grass routes’ system that this enables, empowers local people to make the probiotic fermented food available at prices affordable to poor people. In terms of conferring health benefits, the food matrix appears to have merit for pregnant and non-pregnant women in terms of vaginal and general health [31,32].

There were several limitations to our study. The small sample size was certainly not large enough to detect significant differences in malaria or reliable preterm labor incidence. Also, the lack of contact methods such as cellular phones for some women made follow-up difficult. We also cannot be certain that women who completed the study were fully compliant which may partially explain the lack of changes in the vaginal microbiota in the probiotic group.

In summary, the present proof-of-principle study showed the safe use of encapsulated probiotics in Rwanda in pregnant women, and a degree of receptivity for the concept. The vaginal microbiomes of the women were comparable to those of other countries around the world.

## Supporting information

S1 File CONSORT checklist. (PDF)

S2 File Study protocol. (DOCX)
