# Supplementary material for: Pilot assessment of probiotics for pregnant women in Rwanda
Source: PLoS One. 2018 Jun 18;13(6):e0195081. doi: 10.1371/journal.pone.0195081 (PMC6005520; doi:10.1371/journal.pone.0195081)
Supplement: S2 File — (DOCX) [file pone.0195081.s002.docx]

**Study protocol**

The primary goal of the study is to introduce the concept of probiotics to pregnant women in Rwanda, assess safety and compliance of one month of treatment at various gestational times, and examine whether the therapy impacted the vaginal microbiota.

Recruit pregnant women between the ages of 18 and 55 from the Nyamata District Hospital in Rwanda. Exclude subjects with current infection of gonorrhoea, *Chlamydia*, genital warts, active genital herpes lesions, active syphilis, urinary tract infections, or who are receiving drug therapy that may affect the vaginal microbiome, had unprotected sexual intercourse within the past 48 hours, used a vaginal douche, genital deodorant or genital wipe in past 48 hours, or had taken any probiotic supplement in past 48 hours.

Randomize subjects based on numbering system known only to research student not to clinical staff who are performing the allocation. Subjects will receive capsules of dried *Lactobacillus rhamnosus* GR-1 plus *Lactobacillus reuteri* RC-14 (1 billion viable count per strain) or capsule containing only excipients, by mouth once daily for one month.Treatment (probiotic or placebo) assignment will be matched for malaria exposure in each group. Malaria status will be assessed using the CareStart^TM^ Malaria Rapydtest® kit (Diasys, Berkshire, UK).

Vaginal samples will be collected for metabolome and microbiota analysis at recruitment, one month after treatment and at time of birthing, using dacron polyester-tipped swabs, which will then be frozen within 2 hours of collection and stored at -20 °C or below until shipment to Canada for analysis.

Subjects will return empty vial and verbally confirm compliance. They will be asked to describe any side effects from the treatment.
